# Adjuvant Chemotherapy with Chinese Herbal Medicine Formulas Versus Placebo in Patients with Lung Adenocarcinoma after Radical Surgery: a Multicenter, Randomized, Double-Blind, Placebo-Controlled Trial

**DOI:** 10.1186/s12575-020-00117-5

**Published:** 2020-03-01

**Authors:** Qin Wang, Lijing Jiao, Shengfei Wang, Peiqi Chen, Ling Bi, Di Zhou, Jialin Yao, Jiaqi Li, Liyu Wang, Zhiwei Chen, Yingjie Jia, Ziwen Zhang, Weisheng Shen, Weirong Zhu, Jianfang Xu, Yong Gao, Ling Xu, Yabin Gong

**Affiliations:** 1 Department of Oncology, Yueyang Hospital of Integrated Traditional Chinese and Western Medicine, Shanghai University of Traditional Chinese Medicine, Shanghai, China; 2 Institute of Clinical Immunology, Yueyang Hospital of Integrated Traditional Chinese and Western Medicine, Shanghai University of Traditional Chinese Medicine, Shanghai, China; 3 Department of Thoracic Surgery, Fudan University Shanghai Cancer Center, Shanghai, China; 4 Lung Tumor Clinical Medical Center, Shanghai Chest Hospital, Shanghai Jiaotong University, Shanghai, China; 5 Department of Oncology, First Hospital Affiliated to Tianjin College of Traditional Chinese Medicine, Tianjin, China; 6 Department of Oncology, Changshu No.2 People’s Hospital, Jiangsu, China; 7 Department of Oncology, Jiangyin People’s Hospital, Jiangsu, China; 8 Department of Traditional Chinese Medicine, Ruijin Hospital Affiliated to Shanghai Jiaotong University School of Medicine, Shanghai, China; 9 Department of Oncology, Shanghai Pulmonary Hospital, Tongji University School of Medicine, Shanghai, China; 10 Department of Oncology, Shanghai East Hospital, Tongji University School of Medicine, Shanghai, China

**Keywords:** Lung adenocarcinoma, Chinese herbal medicine formulas, Adjuvant chemotherapy, Adverse events, Disease-free survival

## Abstract

**Background:**

The toxicity and side effects caused by adjuvant chemotherapy (ACT) after radical surgery for lung adenocarcinoma (LAC) lead to early termination frequently. This study was conducted to provide an objective basis for the effect of Chinese herbal medicine formulas (CHMFs) combined with chemotherapy in reducing toxicity and enhancing efficacy of ACT.

**Method:**

From February 17th, 2012 to March 20th, 2015, 233 patients from 7 hospitals diagnosed with LAC in IB~IIIA stage were randomly assigned into ACT + CHMF group (116 patients) and ACT + placebo group (117 patients). CHMF was taken orally until the end of chemotherapy. Chemotherapy-related toxic, side effects were investigated as the primary outcome. Disease-free survival (DFS) and overall survival (OS) were used as the secondary outcome.

**Results:**

At one week following chemotherapy, the incidence of dry mouth, diarrhea and thrombocytopenia significantly decreased in CHMF group (*P* = 0.017, *P* = 0.033, *P* = 0.019, respectively). At two weeks following chemotherapy, fatigue and diarrhea were more obvious in the placebo group (*P* = 0.028, *P* = 0.025, respectively). In addition, patients in the CHMF group showed an increase in median DFS from 37.1 to 51.5 months compared with placebo group although there was no statistical significance (*P* = 0.16). In the stage IB subgroup, the CHMF group had a significantly better DFS (HR (95% CI) = 0.53 (0.28–0.99), *P* = 0.046). There was no significant difference in OS between the groups (*P* = 0.72).

**Conclusion:**

For patients with LAC, ACT combined with CHMF after radical surgery can prolong the DFS time especially in the early stage, and reduces the chemotherapy-related toxic and side effects.

**Trial Registration:**

NCT 01441752. Registered 14 July, 2011.

## Background

Lung cancer is one of the most common causes of cancer-related death all over the world. Non-small cell lung cancers (NSCLC) account for 80% of lung malignancies, of these, roughly 50% are adenocarcinoma, and 18% of Lung adenocarcinoma have an overall 5-year survival [1]. Radical surgery combined with systematic lymphadenectomy is regarded as the primary treatment modality for early stage NSCLC. But even after complete R0 resection, there is still high risk of local recurrence or distant metastases. According to the 8th edition of TNM classification for lung cancer, the 5-year survival rate after surgery is disappointingly low, ranging from 58 to 73% in patients with stage I tumors, 36 to 46% in those with stage II tumors, and only 19 to 24% in those with stage IIIA tumors [2].

In a meta-analysis including 5 largest independent clinical trials, the Lung Adjuvant Cisplatin Evaluation (LACE) collaborative group demonstrated that postoperative cisplatin-based chemotherapy significantly improved survival in patients with NSCLC [3]. The 5-year survival rate was 5.4% higher in the cisplatin-based ACT group than in the group receiving no ACT. Another trial by Adjuvant Navelbine International Trialist Association (ANITA) showed the survival benefit of adjuvant vinorelbine plus cisplatin (NP) versus control in patients with completely resected stage IB~IIIA NSCLC. After a median follow-up of 76 months, median survival was 65.7 and 43.7 months in the chemotherapy group and the observation group respectively [4]. Based on results from these studies and relevant analyses, the guidelines issued by American Society of Clinical Oncology (ASCO) and U.S. National Comprehensive Cancer Network (NCCN) recommend that cisplatin-based two drugs ACT be given to post-surgical NSCLC patients suffering from resectable stage II~IIIA NSCLC or stage IB NSCLC with high risk factors [5, 6].

Although ACT improves the survival of NSCLC patients after surgical resection, it is undeniable that there is a possibility of potential complications or fatal adverse events. In a trial by ANITA, febrile neutropenia occurred in 9% of the patients, with 2% resulting in death [4]. In the JBR.10 trial, hematological toxicity led to death in 2 patients [7]. In a pooled analysis of five trials, the overall incidence of grade 4 toxicity was 32% in the chemotherapy group, and the main reasons for receiving less than the planned number of cycles were patient refusal (35%) and toxicity (34%) [3].

In China, traditional Chinese medicine (TCM) is mainly used clinically in combination with ACT to cope with chemotherapy-related side effects. In addition, TCM used alone in advanced NSCLC patients intolerant to chemotherapy was reported to be able to prevent recurrence and metastasis and prolong the survival time [8, 9]. A retrospective small-sample study was performed in patients with stage II~IIIA NSCLC. It was revealed that compared with the patients receiving ACT alone, those receiving Chinese medicine + ACT enjoyed a longer time without relapse or metastasis [10]. Our previous study showed that Chinese herbal medicines based on syndrome differentiation were able to improve the symptoms, including nausea, fatigue, pain and dry mouth, and also alleviate some chemotherapy-related side effects [11, 12].

The clinical therapeutic benefits of TCM still lack reliable evidence from prospective study. Therefore, this multi-center, prospective, randomized, double-blind trial was designed to demonstrate a higher benefit for CHMF + ACT.

## Materials and Methods

### Study Design and Participants

Participants meeting the following inclusion criteria were enrolled from medical centers: completely resected stage Ib ~ IIIa NSCLC, aged 18~75 years, an Eastern Cooperative Oncology Group performance status (ECOG PS) scale of 0~2 and adequate hematological, biochemical and organ functions. Chemotherapy was started within 6 weeks after surgery and consisted of 4 cycles of treatment with cisplatin/carboplatin and vinorelbine. Any patient meeting the following exclusion criteria was excluded from this clinical study: having other primary malignant tumors; incomplete resection or uncertain to have resection; serious problems of the heart, liver or kidney with severe dysfunction; being pregnant or breast-feeding; having mental or cognitive disorders that would influence judgment of quality of life (QoL) in this study; receiving or about to receive chemotherapy; being participating in other drug trials; allergic to the drug in the study. The study protocol was approved by the institutional review boards (IRBs) of the participating medical centers, the independent National Ethics Committee and Chinese Medicinal Agency, and was registered at Clinical Trials. gov (number: NCT01441752; date: July 14, 2011). All of the participants provided written informed consent before enrolment.

### Procedure

Eligible patients were randomly assigned 1:1 to receive ACT with CHMF or with placebo within 6 weeks after surgery. Central randomization was performed by a clinical research organization (CRO) (Shanghai Clinical Research Center, Shanghai, China; http://www.scrcnet.org). Random numbers were automatically generated by computer referring to pre-configured stratified factors [stage (Ib vs IIa vs IIb vs IIIa), gender (male, female) and the medical center] before administration of ACT. Then the CRO provided the random number to each participating center. The block size and treatment-assignment table were not available to the researchers until the end of the study.

### Syndrome Differentiation Criteria

The CHMFs were prescribed based on syndrome differentiation. Syndrome differentiation criteria were based on “The Guiding Principles of Clinical Research of New Chinese Medicine (trial)” (China Pharmaceutical Technology Publishing House, 2002) and “Shanghai diagnosis and treatment routine of TCM Syndrome”(Shanghai Municipal Health Bureau edit). Three syndromes of TCM are set as follows:

Qi deficiency syndrome is composed of the following symptoms: cough with phlegm, poor appetite, lassitude and weakness, pale and plump tongue; secondary symptoms of spontaneous sweating, loose stool or soft slippery pulse.

Yin deficiency syndrome contains main symptoms of cough with scanty phlegm, dry mouth, reddish tongue; secondary symptoms of night sweating, heartburn and insomnia, low fever, thread and rapid pulse.

Qi and Yin deficiency syndrome contains main symptoms of cough with scanty phlegm, shortness of breath, lassitude and weakness, thirst without the desire to drink; secondary symptoms of spontaneous sweating, night sweating, reddish tongue or tongue with teeth marks, and thread and weak pulse.

The diagnosis can be given with at least two of the main symptoms and one of the secondary symptoms. Each formula was prescribed by one professor who had worked in Longhua hospital for more than 50 years to facilitate clinical research.

Patients who diagnosed with Qi syndrome deficiency, Yin syndrome deficiency received treatment with basic herbs adding YiQi formula and YangYin formula respectively. Patients with both Qi and Yin syndrome deficiency prescribed with basic herbs adding the combination of YiQi and YangYin formula granules. In placebo group, patients have the corresponding placebo granules. The composition of the formulas was shown in Table 1.

**Table 1:** Composition of the basic herbs and added formulas with different syndromes

**Basic Herbs for all patients**					
**Chinese name**	**Chinese name (Pinyin)**	**Pharmaceutical name**	**English name**	**Produced from**	**Dosage (grams)**
**夏枯草**	Xia Ku Cao	*Prunlla vulgaris* L.	Spica Prunellae	Dry spikes	15
**生南星**	Sheng Nan Xing	*Arisaema erubescens* (Wall.) Schott.	Arisaema Rhizoma Arisaematis	Dry rhizoma	30
**蛇六谷**	She Liu Gu	*Amorphophallus konjac* K.Koch	Rhizoma Amorphophalli	Dry tubers	30
**山慈菇**	Shan Ci Gu	*Cremastra appendiculata* (D.Don) Makino	Pseudobulbus Cremastrae Seu Pleiones	Dry pseudobulb	15
**泽漆**	Ze Qi	*Euphorbia helioscopia* L.	Euphorbiae Helioscopiae	Herba	15
**石上柏**	Shi Shang Bai	*Selaginella Doederleinii* Hieron	Selaginella Doederleinii	Herba	30
**石见穿**	Shi Jian Chuan	*Salvia chinensis* Benth.	Salviae Chinensis	Herba	30
**重楼**	Chong Lou	*Paris polyphylla* Smith var. chinensis (Franch.) H.Hara	Rhizoma Paridis	Dry rhizoma	15
**海藻**	Hai Zao	*Sargassum*	Seaweed	Leaves	15
**瓜蒌皮**	Gua Lou Pi	*Trichosanthes kirilowii* Maxim.	Pericarpium Trichosanthis	Pericarp	15
**猫爪草**	Mao Zhua Cao	*Ranunculus ternatus* Thunb.	Radix Ranunculus Ternati	Herba	15
**生牡蛎**	Sheng Mu Li	*Concha Ostreae*	Oyster Shell	Shell	30
**大枣**	Da Zao	*Ziziphus jujuba* Mill.	Fructus Jujubae	Fruit	9
**For Qi syndrome deficiency, add Yiqi formula:**					
**Chinese name**	**Chinese name(Pinyin)**	**Pharmaceutical name**	**English name**	**Produced from**	**Dosage (grams)**
**黄芪**	Huang Qi	*Astragalus membranaceus* Fisch. ex Bunge	Milkvetch Root Radix Astragali	Dry rhizoma	30
**党参**	Dang Shen	*Codonopsis pilosula* (Franch.) Nannf.	Codonopsis Radix	Dry rhizoma	9
**白术**	Bai Zhu	*Atractylodes macrocephala* Koidz.	Atractylodis Macrocephalae Rhizoma	Dry rhizoma	12
**茯苓**	Fu Ling	*Poria cocos* (Schw.) Wolf.	Indian Bread Poria	Dry rhizoma	15
**淫羊藿**	Yin Yang Huo	*Epimedium brevicornu* Maxim.	Epimedii Folium	Herba	15
**葫芦巴**	Hu Lu Ba	*Trigonella foenum-graecum* L.	Common Fenugreek Seed Semen Trigonellae	Dry seed	15
**补骨脂**	Bu Gu Zhi	*Psoralea corylifolia L.*	Fructus Psoraleae	Fruit	12
**For Yin syndrome deficiency, add Yangyin formula:**					
**Chinese name**	**Chinese name(Pinyin)**	**Pharmaceutical name**	**English name**	**Produced from**	**Dosage (grams)**
**北沙参**	Bei Sha Shen	*Glehnia littoralis* (A.Gray) F.Schmidt ex Miq.	Coastal Glehnia Root	Dry rhizoma	30
**南沙参**	Nan Sha Shen	*Adenophora stricta* Miq.	Fourleaf Ladybell Root	Dry rhizoma	30
**天冬**	Tian Dong	*Asparagus cochinchinensis* (Lour.) Merr.	Cochinchinese Asparagus Root	Dry rhizoma	15
**麦冬**	Mai Dong	*Ophiopogon japonicus* (Thunb.) Ker Gawl.	Dwarf Lilyturf Tuber	Dry rhizoma	15
**百合**	Bai He	*Lilium brownii* var. viridulum Baker	Lilii Bulbus	Scale leaf	15
**女贞子**	Nv Zhen Zi	*Ligustrum lucidum* W.T.Aiton	Fructus Ligustri Lucidi	Fruit	12

### Pharmaceutical Ingredients

Three formulas (batch numbers: 1106070, 1106011, 1106041) were dynamic cycle extracted, then concentrated and spray-dried to make granules by Jiangyin Tianjiang Pharmaceutical Co. Ltd. (Jiangsu, China).

In conformity with Good Clinical Practice (GCP), in double-blind clinical trials, the investigational drug and placebo should be identical in appearance (color, viscosity, hardness, and other physical properties), smell, packaging, labeling, and other characteristics. It is difficult to define a placebo in Chinese herbal medicine where all-natural substances are potentially therapeutic. Therefore, the raw materials for the placebo including 10% of CHMF, and food color, artificial flavors. Ingredient of food color and artificial flavors can be found in our previous study [12]. At this concentration, the placebo was reported having no physiological activity [13, 14].

### Intervention

Patients received continued ACT (vinorelbine, 25 mg/m^2^, on days 1 and 8, twice every 21 days and cisplatin 75 mg/ m^2^, on day 1, once every 21 days or carboplatin AUC (area under the curve) = 5, on day 1, once every 21 days). Meanwhile, patients enrolled in the study received treatment with CHMFs or matched placebo twice a day until the end of chemotherapy.

The CHMF and placebo granules were administered on the first day after chemotherapy. The medicine was dissolved in 160 mL of warm water for drinking at 9:30 a.m. and 15:00 p.m. All procedures were carried out under the supervision of clinical research pharmacists. The processed herbs and the granules were prepared in compliance with Good Manufacturing Practice (GMP).

Chest computed tomography scan was carried out every 2 cycles to evaluate the clinical response. Within the first 2 years following the end of chemotherapy, a chest CT scan was performed every 2 months, then from the third year every 6 months [15]. Blood chemistry, hematology, concomitant medications, and adverse events were assessed on day 1 of each 21-day cycle. Adverse events including toxicity and side effects were reported according to Common Terminology Criteria for Adverse Events V3.0 (CTC AE) issued by National Cancer Institute (NCI) (http://ctep.info.nih.gov). All of the unexpected responses possibly or definitely related to the study were reported. In case of serious adverse events (SAEs), treatment was stopped immediately and appropriate treatment was provided. Patients with disease progression continued to be assessed every 3 months.

### Statistical Analysis

All of the statistical analyses on data were performed using the software SPSS 18.0. Chi-square test was employed for baseline analysis and analysis on enumeration data, and rank-sum test was utilized for analysis on ordered data (NCI-CTC classification of AEs). The DFS time referred to the time interval between the date of enrolment and the date of disease recurrence or metastasis, the data were analyzed with log-rank test, the DFS and OS curves were estimated using the Kaplan–Meier method, the 95% confidence interval (CI) was calculated using the Greenwood formula, and COX regression analysis was employed for stratified analysis. All of the data were eligible random data obtained from patients in the full-analysis set.

## Results

**Fig. 1:**
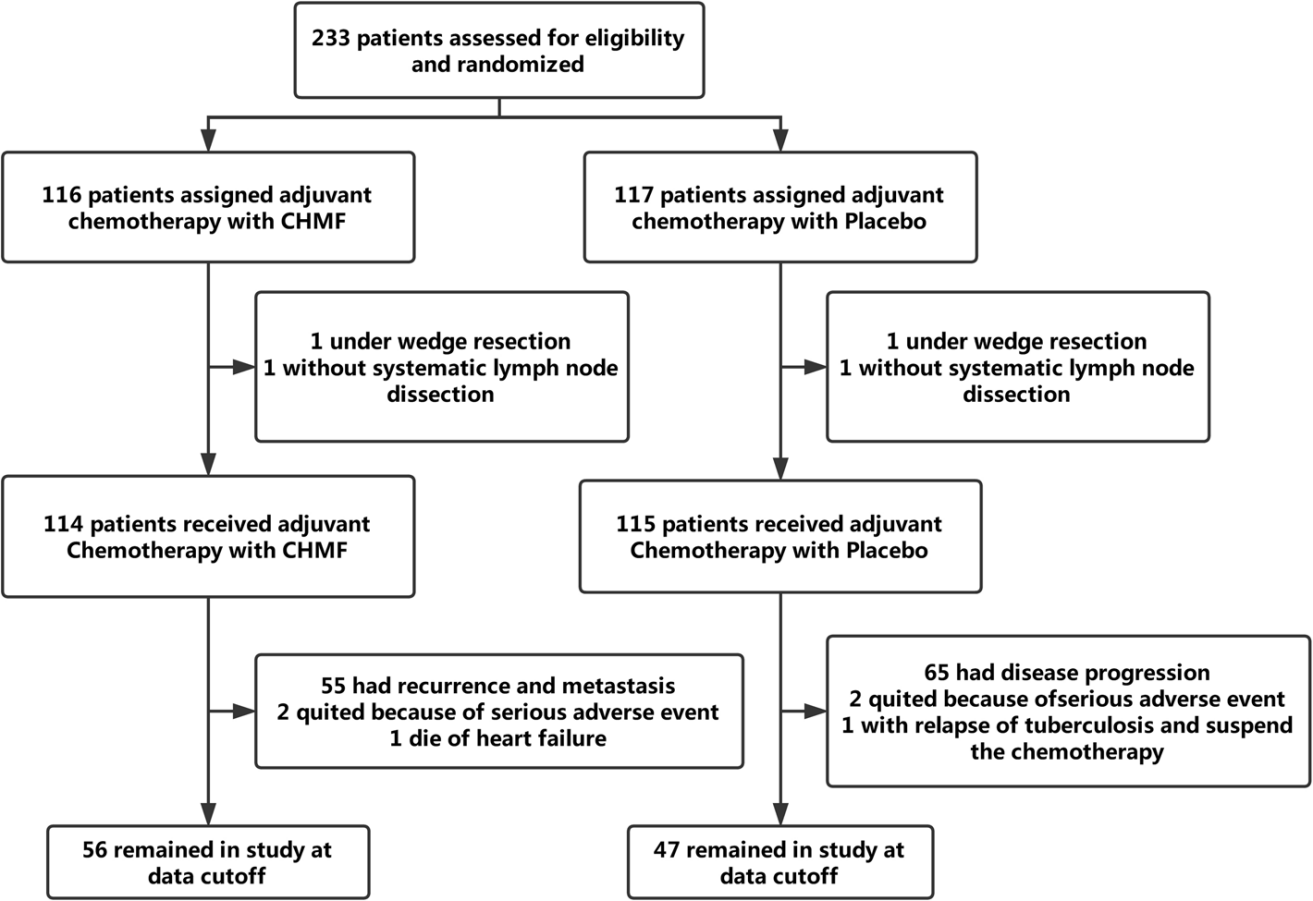
Study profile. Data cutoff was April 16, 2018. CHMF, Chinese herbal medicine formula

From February 17th, 2012 to March 20th, 2015, a total of 233 patients with stage IB-IIIA lung adenocarcinoma after radical surgery were selected from 7 hospitals. Among them, 116 patients were randomly assigned into the ACT + CHMF group, and 117 patients were assigned into the ACT + placebo group. In each group, there were patients excluded from analysis because of the performance of wedge resection and without systematic mediastinal lymphadenectomy. At the time of data cutoff (April 16th, 2018), a total of 55 patients experienced recurrence and metastasis in the chemotherapy + CHMF group. Two patients experienced serious side effects and withdrew from study and one patient died from heart failure. In the chemotherapy +placebo group, a total of 65 patients experienced recurrence and metastasis, two patients withdrew because of serious side effects and one patient discontinued chemotherapy for relapse of tuberculosis. These patients were included in the intention-to-treat efficacy analysis as shown in Fig. 1. There was no significant difference in age, sex, Eastern Cooperative Oncology Group (ECOG) Performance Status, smoking history, resection procedure, video assistance or not, p-TNM stage, chemotherapy regimens and TCM syndrome between two groups. See Table 2.

**Table 2:** Baseline characteristics and demographics of the population received adjuvant treatment. Data are expressed as numbers (percentage) or mean (range)

	Adjuvant Chemo + CHMF	Adjuvant Chemo + Placebo	*P*-value
	(*n* = 114)	(*n* = 115)	
Gender, n (%)			0.596
Male	57 (50%)	61 (53%)	
Female	57 (50%)	54 (47%)	
Age	59.4 ± 7.4	59.6 ± 7.5	0.861
ECOG PS, n (%)			0.99
0	2 (1.8%)	2 (1.7%)	
1	111 (97.4%)	112 (97.4%)	
2	1 (0.8%)	1 (0.9%)	
Smoking history, n (%)			0.224
Smoked	40 (35.1%)	50 (43.5%)	
Never smoke	74 (64.9%)	65 (56.5%)	
Resection procedure, n (%)			0.333
Lobectomy	108 (94.7%)	112 (97.4%)	
Sublobectomy	6 (5.3%)	3 (2.6%)	
Video-assaciated, n (%)			0.689
Yes	64 (56.1%)	68 (59.1%)	
No	50 (43.9%)	47 (40.9%)	
pTNM stage, n (%)			0.998
Ib	55 (48.2%)	56 (48.7%)	
II	22 (19.3%)	22 (19.1%)	
III	37 (32.5%)	37 (32.2%)	
Chemo regimens, n (%)			0.688
NP	48 (42.1%)	45 (39.1%)	
NC	66 (57.9%)	70 (60.9%)	
TCM syndrome, n (%)			0.862
Qi deficiency	55 (56.1%)	58 (50.4%)	
Yin deficiency	10 (8.8%)	8 (7.0%)	
Qi-Yin deficiency	49 (35.1%)	49 (42.6%)	

Abbreviations: Chemo, chemotherapy; *CHMF* Chinese herbal medicine formula, *ECOG PS* Eastern Cooperative Oncology Group Performance Status, *NP* Vinorelbine plus cisplatin, *NC* Vinorelbine plus Carboplatin

### Effect of CHMF Addition to ACT on Toxicity and Side Effects

**Fig. 2:**
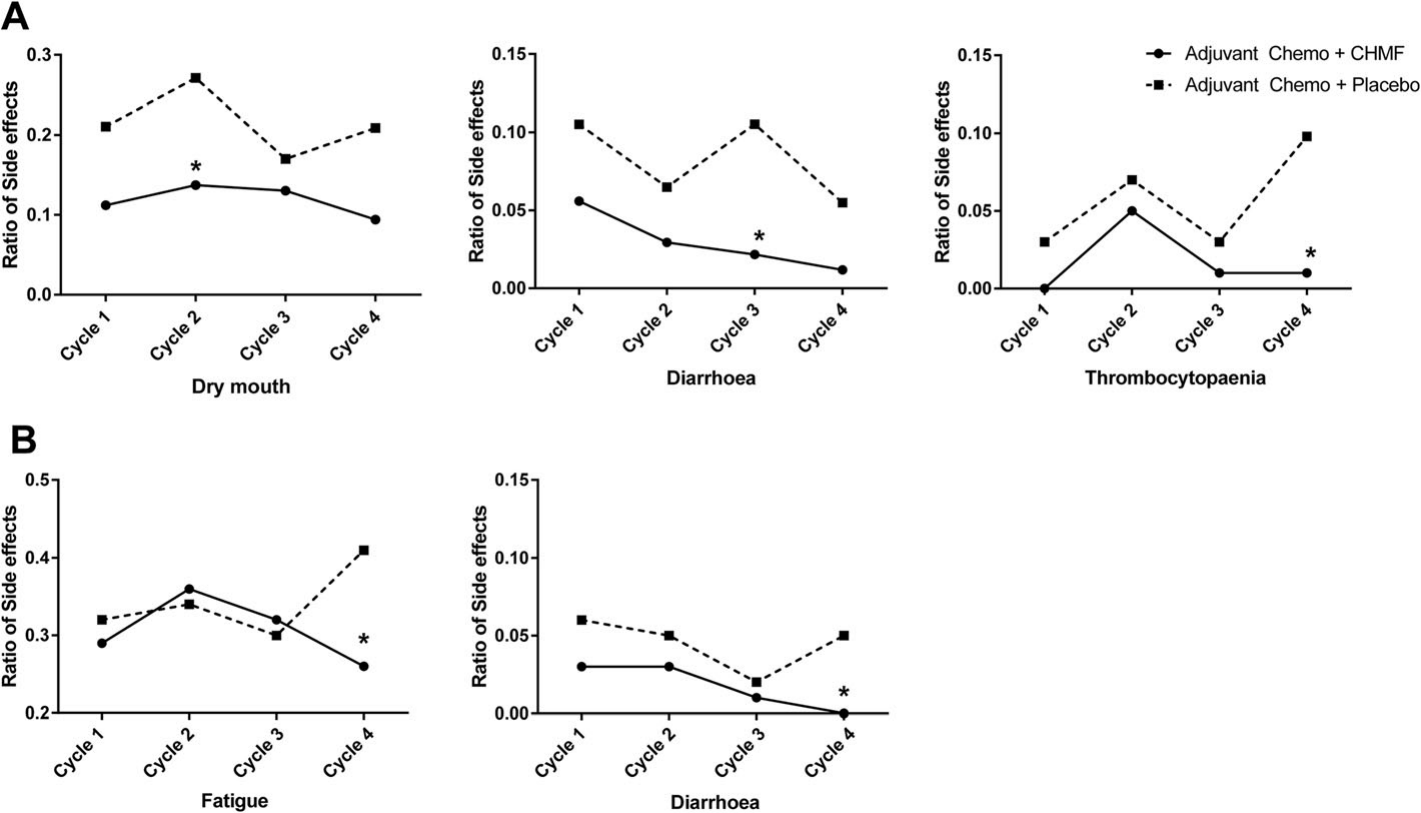
Figures present adverse events which significantly decreased at one week (**a**) and two weeks (**b**) following chemotherapy in the CHMF group (solid line) compared with the placebo group (dotted line). * stands for *P<*0.05

Adverse events including toxicity and side effects were reported according to CTC AE issued by NCI (https://ctep.cancer.gov/protocolDevelopment/Electronicapplications/docs/ctcaev3.pdf). The five most common side effects caused by regimens occurring in > 40% of patients were fatigue, loss of appetite, nausea, leucopenia and vomiting. At 1 week after last course of treatment, the incidence of these side effects decreased in the CHMF group. The incidence of grade 3–4 serious adverse events (SAEs) was27.2% (31/114) and 30.4% (35/115) respectively in the CHMF group and the placebo group respectively. See Table 3. In addition, the incidence of dry mouth, diarrhea and thrombocytopenia decreased significantly in the CHMF group compared with the placebo group, with 24.6% vs. 39.1% (1 week after chemotherapy at cycle 2, *P* = 0.017), 7.9% vs. 17.4% (1 week after chemotherapy at cycle 3, *P* = 0.033) and 5.3% vs. 13% (1 week after chemotherapy at cycle 4, *P* = 0.019) in the two groups respectively. Also, at 2 weeks after chemotherapy at cycle 4, fatigue and diarrhea were significantly reduced in the CHMF group than in the placebo group (*P* = 0.028, 0.025, respectively). See Fig. 2.

**Table 3:** Adverse events during the adjuvant treatment phases. Values are expressed as numbers (percentage). Table presents grade I to IV adverse events in all patients during the adjuvant treatment phases. Adverse events are listed in descending order of frequency in the total patient population

	Adjuvant chemo + CHMF (*N* = 114) n (%)			Adjuvant chemo+ Placebo (*N* = 115) n (%)		
	Grade 1/2	Grade 3/4	Total	Grade 1/2	Grade 3/4	Total
Fatigue	90 (78.9%)	1 (0.8%)	91 (79.7%)	89 (77.45%)	0	89 (77.45%)
Loss of appetite	88 (77.2%)	0	88 (77.2%)	94 (81.7%)	0	94 (81.7%)
Nausea	77 (67.5%)	1 (0.8%)	78 (68.3%)	77 (67%)	0	77 (67%)
Leucopenia	49 (43.6%)	8 (7%)	57 (50.6%)	40 (34.8%)	15 (13%)	55 (47.8%)
Vomiting	43 (37.3%)	6 (4.4%)	49 (41.7%)	47 (40.9%)	1 (0.8%)	48 (41.7%)
Elevated TB	37 (32.5%)	1 (0.8%)	38 (33.3%)	40 (34.8%)	0	40 (34.8%)
Constipation	37 (32.5%)	1 (0.8%)	38 (33.3%)	39 (33.9%)	0	39 (33.9%)
Neutropenia	34 (29.8%)	11 (9.7%)	45 (39.5%)	37 (32.2%)	17 (14.8%)	54 (47%)
Anaemia	29 (25.4%)	0	29 (25.4%)	28 (24.3%)	1 (0.8%)	29 (25.1%)
Dry mouth	28 (24.6%)	0	28 (24.6%)	45 (39.1%)	0	45 (39.1%)
Pain	28 (24.7%)	0	28 (24.7%)	37 (32.2%)	0	37 (32.2%)
Alopecia	13 (11.4%)	0	13 (11.4%)	14 (12.2%)	0	14 (12.2%)
ALT/AST increased	12 (10.5%)	1 (0.8%)	13 (11.3%)	15 (11.3%)	0	15 (11.3%)
Elevated GGT	11 (9.6%)	2 (1.8%)	13 (11.4%)	9 (7.8%)	1 (0.8%)	10 (8.6%)
Diarrhoea	9 (7.9%)	0	9 (7.9%)	20 (17.4%)	0	20 (17.4%)
Thrombocytopaenia	6 (5.3%)	0	6 (5.3%)	15 (13%)	1 (0.8%)	16 (13.8%)
Elevated CRE	4 (3.5%)	0	4 (3.5%)	1 (0.9%)	0	1 (0.9%)
Weight-decrease	4 (3.5%)	0	4 (3.5%)	7 (6.1%)	0	7 (6.1%)
Rash	3 (2.6%)	0	3 (2.6%)	2 (1.7%)	0	2 (1.7%)
Arhythmia	2 (1.8%)	0	2 (1.8%)	2 (1.7%)	1 (0.8%)	3 (2.5%)
Pruritus	2 (1.8%)	0	2 (1.8%)	4 (3.5%)	0	4 (3.5%)
Elevated ALP	1 (0.8%)	0	1 (0.8%)	1 (0.9%)	0	1 (0.9%)

### Effect of CHMF Addition to ACT on the DFS

**Fig. 3:**
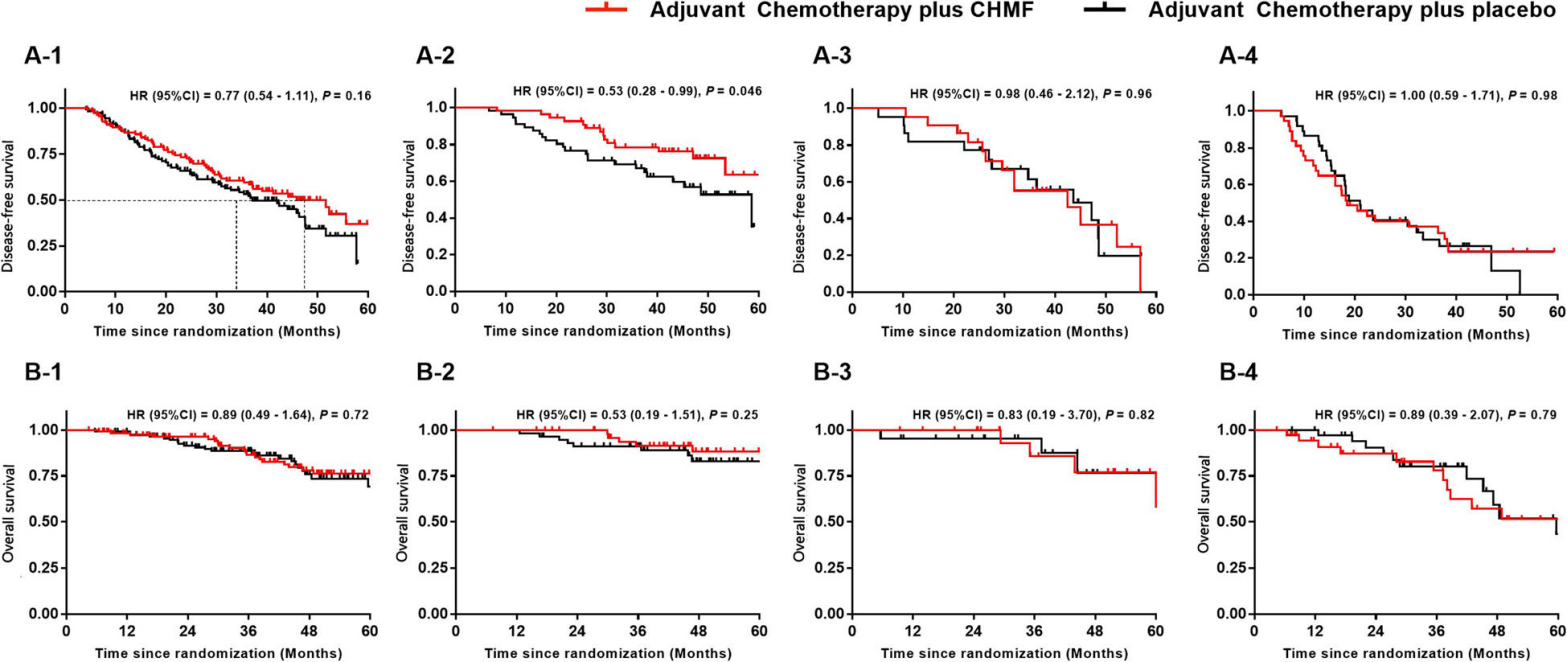
Progression-free survival (A-1) and overall survival (B-1) of all patients and patients with stage Ib-IIIa (A/B2–4). Kaplan-Meier estimates of progression-free survival and overall survival as assessed by investigators in populations received adjuvant treatment between CHMF (Red) and placebo (Black) arms. *P* values were calculated using a two-sided log-rank test. HR, Hazard ratio; CI, confidence interval; CHMF, Chinese herbal medicine formula

The secondary endpoint of the study was DFS, defined as the time interval between the date of enrolment and the date of disease recurrence or metastasis. After a median follow-up of 53.6 months (IQR 5.6–75.1). Kaplan-Meier survival analysis showed a median DFS time of 51.5 and 37.1 months in the CHMF group and the placebo group respectively [HR = 0.77 (0.54–1.11), *P* = 0.16]. Subgroup analysis was conducted by TNM stage. For patients with disease at stage II to IIIa, there was no significant difference in DFS between the groups. However, in patients with stage IB lung adenocarcinoma, Kaplan-Meier survival analysis showed a significant effect of ACT plus CHMF on DFS [HR = 0.53 (0.28–0.99), *P* = 0.046]. See Fig. 3 A1–4.

### Effect of CHMF Addition to ACT on the OS

OS was defined as the time interval between the date of enrolment and the date of death by any cause. Kaplan-Meier survival analysis did not show a statistically significant improvement in OS in the CHMF group [HR = 0.89 (0.49–1.64), *P* = 0.72]. At the end of the follow-up period, two groups did not reach the median survival time. Subgroup analysis also showed a similar benefit in the groups according to TNM stage. See Fig. 3 B1–4.

## Discussion

According to the NCCN clinical practice guidelines in oncology, cisplatin-based two drugs ACT was given to post-surgical NSCLC patients with resectable stage II~IIIA NSCLC or stage IB NSCLC with high-risk factors [6]. To our knowledge, this is the first multicenter, randomized, and double-blind trial to demonstrate the advantages of ACT combined with CHMF in improving DFS.

The ANITA initiated a randomized phase III trial in patients with completely resected stage IB, II, and IIIA NSCLC in 2006, and found a survival benefit for ACT after a median follow-up of 76 months. The analysis showed a median survival of 65.7 months in the chemotherapy group and 43.7 months in the observation group. In our trail, after a median follow-up of 53.6 months, analysis did not show a statistically significant improvement in OS in the CHMF group [HR = 0.89 (0.49–1.64), *P* = 0.72]. The groups did not reach the median survival time, possibly because of the improved selectivity of cancer drugs for patients when recurrence and metastasis occurred. After chemotherapy, CHMF treatment might also be required in the placebo group and targeted therapies were often given, which were the confounding factors for OS assessment.

In the current study, it was found that there was an improvement of DFS in the CHMF group without statistical significance [HR = 0.77 (0.54–1.11), *P* = 0.16]. The subset analysis of stage showed that ACT + CHMF was able to obviously prolong the DFS time in patients with stage IB disease [HR = 0.53 (0.28–0.99), *P* = 0.046]. The DFS time was not significantly different between the groups in patients with stage II and IIIA disease.

According to the 7th TNM classification for lung cancer, the 5-year survival rate of stage IB lung cancer is 66%, significantly lower than that of stage IA cancer (82%) [2]. Stage IB NSCLC patients with high-risk factors including poor differentiated tumor, tumor size > 4 cm, visceral pleural invasion, vascular invasion and wedge resection [6] are advised to receive ACT based on a prospective randomized clinical trial [16]. In addition to the above factors, pathological classification also has guiding significance for patients to receive ACT or not. Solid/micropapillary patterns were associated with poor prognosis [17]. The predominant micropapillary/solid pattern in patients with stage IB lung adenocarcinoma who received ACT had obviously lower cumulative incidences of lung cancer-related death and recurrence [18, 19]. Therefore, CHMF alone or in combination with ACT has potential advantages in patients with stage IB lung adenocarcinoma after radical surgery.

In a previous study, it was shown that chemotherapy could lower the quality of life in a short term, which was mainly associated with fatigue, nausea and vomiting [4]. Adjuvant vinorelbine plus cisplatin had an acceptable level of toxicity and prolonged disease-free survival and overall survival among patients with completely resected NSCLC. Analysis of adverse events indicated that anemia, neutropenia, fatigue, loss of appetite, and vomiting were frequent in the patients with incidence over 50% [4, 7].

Quality of life assessment was performed in 359 patients, and the overall incidence of neutropenia was no more than 50%, with 39.5 and 47% in the CHMF group and the control group respectively. In this trial, the most frequent side effects caused by NP/NC ACT included fatigue, loss of appetite, nausea, leucopenia and vomiting. The incidence of grade 3–4 toxic and side effects was 27.2 and 30.4% in the ACT + CHMF group and the ACT + placebo group respectively. The incidence of grade 3–4 leucopenia and neutropenia was 7% vs. 13 and 9.7% vs. 14.8% respectively, without statistical significance (*P* = 0.129; *P* = 0.236). These results were similar to those in patients with stage IB~IIIA NSCLC that received NP/NC ACT [4]. In the ANITA trial, the incidence of agranulocytosis was lower than 85% in the chemotherapy group.

Nevertheless, the incidence of grade 3–4 vomiting was higher in the ACT + CHMF group than in the ACT + placebo group (4.4% vs. 0.8%, *P* = 0.053), which may be associated with the fact that Chinese medicines were taken orally immediately after chemotherapy and helps to determine the best time to take Chinese medicines. Meanwhile, we separately compared the toxic and side effects at 1 and 2 weeks after chemotherapy. In comparison of side effects caused by chemotherapy at 1 week after chemotherapy, dry mouth was more remarkable in the placebo group than in the CHMF group at cycle 2 (*P* = 0.017); diarrhea was more evident in the placebo group than in the CHMF group at cycle 3 (*P* = 0.033); and thrombocytopenia was more markedly in the placebo group than in the CHMF group at cycle 4 (*P* = 0.019). At 2 weeks after chemotherapy, fatigue and diarrhea were more obvious in the placebo group than in the 18MF group at cycle 4 (*P* = 0.028, *P* = 0.025). This indicated that Chinese medicines alleviated the symptoms (diarrhea, thrombocytopenia and fatigue) mostly at cycles 3 and 4, consistent with the characteristics of Chinese medicines that they act slowly with sustained effect.

Our previous study revealed that Yin deficiency and Qi deficiency of the lung were the major types of syndrome in lung cancer. Yin deficiency of the lung was constantly present in the disease, more remarkable at the early stage, which was associated with the physiological property of the lung, i.e. preference for moistness and intolerance to dryness [20]. Therefore, treatment nourishing Yin was given in the present study to improve the symptom dry mouth after chemotherapy. Meanwhile, progress-free survival (PFS) was analyzed in patients with different types of syndrome, and no difference was observed in prognosis. In early-stage lung adenocarcinoma, intervention with Chinese medicines + ACT was more effective in regulating imbalance of Yin and Yang and improving the symptoms, hence playing a synergistic role in fighting against tumor.

The limitation of this study lies in the fact that different prescriptions were used for different types of syndrome and each of the prescriptions was comprised of multiple ingredients, demanding further study.

## Conclusion

In conclusion, Chinese herbal medicines enable ACT to prolong the DFS time in patients with stage IB lung adenocarcinoma, and reduce the toxic and side effects caused by chemotherapy, including dry mouth, fatigue, diarrhea and thrombocytopenia.

## Data Availability

To support the results reported, data and materials can be found on the website https://www.clinicaltrials.gov/ct2/show/NCT01441752?term=01441752&rank=1.

## Abbreviations

ACT: Adjuvant chemotherapy
ANITA: Adjuvant Navelbine International Trialist Association
ASCO: American Society of Clinical Oncology
CHMFs: Chinese herbal medicine formulas
CI: Confidence interval
CRO: Clinical research organization
DFS: Disease-free survival
ECOG: Eastern Cooperative Oncology Group
GCP: Good Clinical Practice
GMP: Good Manufacturing Practice
IRBs: Institutional review boards
LAC: Lung adenocarcinoma
LACE: Lung Adjuvant Cisplatin Evaluation collaborative group
NCCN: National Comprehensive Cancer Network
NCI: National Cancer Institute
NSCLC: Non-small cell lung cancers
OS: Overall survival
PS: Performance status
QoL: Quality of life
SAEs: Serious adverse events
TCM: Traditional Chinese medicine
